# Vaccinations for Colorectal Cancer: Progress, Strategies, and Novel Adjuvants

**DOI:** 10.3390/ijms20143403

**Published:** 2019-07-11

**Authors:** Stephen Jiang, David Good, Ming Q. Wei

**Affiliations:** 1 School of Medical Science and Menzies Health Institute Queensland, Gold Coast campus, Griffith University, Southport, QLD 4222, Australia; 2 School of Allied Health, Australian Catholic University, Banyo, QLD 4014, Australia

**Keywords:** cancer vaccine, adjuvant, cytokine, galectin inhibitor, colorectal cancer, immune checkpoint inhibitor, regulatory T cell, immunotherapy

## Abstract

Although cancer is a leading cause of death, significant breakthroughs have been made in its treatment in recent years. In particular, increasingly effective cancer vaccines are being developed, including some for colorectal cancer. There are also currently a variety of compounds that can act as adjuvants, such as signalling molecules called cytokines. Other adjuvants target and inhibit the specific mechanisms by which cancers evade the immune system. One of them is a galectin inhibitor, which targets galectins—proteins produced by cancer cells that can cause the death of immune cells. Likewise, immune checkpoint inhibitors affect immune checkpoints—natural host proteins that usually control inflammation but can be exploited by cancers to weaken the body’s defences. Equally, regulatory T cells may contribute to the progression of cancer by inhibiting the functions of other T cells. The main advantages of cancer vaccines include their low toxicity and their ability to strengthen the immune system. Nevertheless, significant limitations include their slow effects and their inability to treat cancer at times due to immunosuppression. Ultimately, ongoing trials provide hope for the development of more effective methods of immunotherapeutic inoculation that can target a greater variety of cancers.

## 1. Introduction

Cancer is one of the leading causes of death in modern society, and the death rate continues to increase drastically due to ageing populations [1]. The International Agency for Research on Cancer has produced the GLOBOCAN 2018, which estimates that there were 18.1 million new cancer cases across the globe, which resulted in 9.6 million deaths [1]. The most frequently encountered types of cancer are lung cancer, breast cancer, prostate cancer and colorectal cancer (CRC) [1]. In particular, lung cancer comprises 11.6% of cases and causes 18.4% of cancer deaths [1]. 

The main reason why cancer is of such high concern is the difficulty associated with its treatment. This is due to cancer not being one disease, but rather a collection of different non-communicable diseases affecting various parts of the body [2]. Each tumour arises from the accumulation of mutations that are present in different combinations in individuals, and thus, it is challenging to create one treatment that can cure all types of cancer [3]. These alarming statistics serve to reinforce the impact that these conditions have on the lives of those affected and their loved ones. They also emphasise the need for the development of more effective cancer treatments, such as cancer vaccines and immunotherapy. 

Immunotherapy increases the strength of immune systems to treat cancer [4]. There are numerous different types of immunotherapy, including some involving interferons, monoclonal antibodies and inoculation [4]. Although many cancer vaccines prevent cancer from occurring, there are also therapeutic ones that can aid in its treatment, offering hope to those already affected [4,5]. Furthermore, a large number of substances can also be used to increase the effectiveness of immunisation, and these are referred to as adjuvants [4]. 

Thus, this review aims to showcase the current status of cancer vaccines and the further improvements that can be made through the utilisation of adjuvants. It investigates a wide range of adjuvants and evaluates their effectiveness, with emphasis on the treatment of CRC. It is hypothesised that adjuvants, when combined with cancer vaccines, significantly enhance the immune system’s ability to fight cancer synergistically. Synergism occurs when the combined effect of two substances is greater than their individual results [4]. This means that when multiple adjuvants are combined, the resulting treatment is significantly better [4]. 

The long histories of vaccines and adjuvants need to be investigated first to understand their progression and development over the years [6]. In the 18th century, Edward Jenner demonstrated that inoculation techniques could help protect individuals against smallpox without transmitting the disease [6]. Subsequently, several advances occurred in the early 20th century when vaccination programs were introduced and toxoid vaccines were produced [6]. Adjuvants have a shorter history in comparison with cancer vaccines [6]. During the 1920s, Gaston Ramon discovered that substances could enhance the effects of vaccines while he was developing vaccines for diphtheria and tetanus [6]. Around the same time, Alexander Glenny discovered that aluminium is an adjuvant [6]. For approximately 70 years after 1932, aluminium was the only adjuvant used in licensed human vaccines [6]. Although it is commonly used, aluminium is an experimentally demonstrated neurotoxin, and there is still a limited understanding of its mechanism of action [6]. Another example of an early adjuvant is a water in oil emulsion (Freund’s Incomplete Adjuvant) developed in 1930, named after Jules T. Freund [6]. However, it produced too many adverse reactions [6]. The problems associated with earlier adjuvants emphasise the need for the discovery of more effective substances to produce stronger vaccines. This review will explore how these treatment methods can be applied to CRC.

Colorectal carcinomas (CRC) can occur in the rectum, where they are called rectal cancer [7]. They can also start in the colon, resulting in colon cancer [7]. Precancerous polyps are the cause of some of these cancers [8]. Polyps are abnormal growths, which most commonly occur in the inner lining of the colon [8]. Some polyps change into cancer, while others do not [8]. They may be flat in appearance or resemble mushroom-like stalks [8]. 

The symptoms of CRC include stomach cramps, pain during bowel movements and rectal bleeding, which can lead to the presence of blood in faeces [9]. However, numerous CRC patients may be initially asymptomatic, creating difficulties in diagnosis [9].

Individuals 50 years or older are encouraged to be screened for colorectal cancer [10]. These screening tests are useful and have minimal side effects [11]. Colonoscopies combine screening with prevention and the removal of polyps immediately after detection [11]. However, only 40% of CRCs are diagnosed when they are still localised [12]. This is especially problematic since the five-year survival rate decreases tremendously after metastasis occurs, from 90% to 13% [12]. 

There is a strong genetic factor contributing to the presence of CRC, resulting in individuals being more at risk if they have relatives with the condition [13]. Inflammatory bowel diseases can also increase the risk of CRC [13]. Moreover, the development of CRC is also influenced by the environment [11]. This emphasises the importance of healthy lifestyle habits, including adequate exercise and the consumption of foods that are high in fibre [11]. Smoking, alcohol and discretionary, high-fat foods should all be avoided [11].

Therapy for CRC exists in the form of surgery and chemotherapy [14]. Although these techniques have improved tremendously over the years, there are still concerns regarding their side effects and efficiency [14]. Cancer vaccines and adjuvants offer a potential solution to this problem [14]. For example, it has been demonstrated that immune checkpoint inhibitors have been highly effective against certain cancer types, including CRC [14].

This discussion delves deeper into the roles of cytokines, especially interferons, in aiding immune cell communication and cancer antigen expression. Having discussed the immune response against cancer, the factors that contribute to cancer survival are presented. These include galectins, antigenic variation and the ability for cancer cells to take control of immune checkpoints and regulatory T cells. In the following section, different types of cancer vaccines, such as tumour cell, antigen, dendritic cell (DC) and allogeneic whole cell vaccines are introduced. Ultimately, the final section amalgamates the information presented in all the topics by establishing how signalling molecules, monoclonal antibodies, phosphorylated adjuvants and galectin inhibitors can all be utilised as adjuvants to strengthen cancer vaccines.

## 2. Signalling Molecules of the Immune System

The purpose of vaccines is to create a stronger immune response against cancer in the body [4]. This response is a complex process that involves various cells from different lines of defence [15]. Cancer cells produce abnormal proteins that are displayed as surface antigens on their major histocompatibility complex (MHC) class I molecules, which are then recognised by immune cells as being non-self and foreign [16]. While MHC class II molecules are usually only found on antigen presenting cells, MHC class I molecules are found on all nucleated cell surfaces and present endogenous antigens to the immune system [17]. Antigen-presenting cells of the innate immune response, such as DCs and macrophages, phagocytose cancer cells before displaying their specific antigens on their MHC class II proteins [17]. These antigens are then recognised by cells of the adaptive immune response such as T helper cells [18]. This initiates the targeted destruction of cancer cells, demonstrating the interplay between cells of the second and third line of defence [17]. Naïve T helper cells proliferate and differentiate with the help of antigen-presenting cells, producing T helper cells and memory cells [17]. The resulting T helper cells can then produce cytokines that not only activate macrophages but also stimulate the proliferation and differentiation of other cells such as B cells and cytotoxic T cells [17]. Cytotoxic T cells are part of cell-mediated immunity and are mainly involved in the destruction of virus-infected and cancer cells, thus being of particular importance when discussing cancer immunology [17].

The cooperation of numerous cells requires effective communication between them, and signalling molecules called cytokines are needed to facilitate this [6]. Stimuli can cause cells to release cytokines, which bind to complementary receptors on target cells to activate genes and produce various biological effects [6]. For example, cytokines can increase inflammation and the division of white blood cells, which generate a stronger immune response against cancer [6]. When an infection occurs, the concentration of cytokines in serum can increase a thousand-fold, demonstrating their importance as integral components of immune responses [6]. 

A previous experiment established the effectiveness of the cytokine tumour necrosis factor-α, interleukin-1 and interleukin-6 in inhibiting the progression of lung cancer [6]. Lung cancer cell lines were incubated with the cytokines before their metabolic activity was analysed via an MTT assay [6]. Results showcased how all three of the cytokines increased the expression of cancer cell antigens [6]. This caused the immune system to recognise cancer cells more efficiently and thus produce a stronger response [6]. Furthermore, tumour necrosis factor-α had the additional function of decreasing their proliferation due to its ability to cause necrosis or apoptosis in cells, thereby reducing the number of cancer cells in the body [6].

Interferons are cytokines that are also important in combating cancer [19]. By binding to the cell surface receptors of target cells, a signal cascade is initiated [19]. It eventually increases the production of transcription factors that allow the transcription of genes involved in reducing cell division [20]. This suppresses the growth of tumours, similarly to the function of tumour necrosis factor-α [20]. Interferons also serve to increase the presentation of cancer cell antigens, which is explained further in Topic 4 in conjunction with cancer vaccines [21]. For example, the increased expression of MHC class 1 is associated with IFN-γ [21]. 

In 2017, an experiment was performed to investigate the effects of interferon-α (IFNα) on the stimulation of anti-tumour responses [22]. The researchers observed whether the substance increased the expression of cancer stem cell (CSC) markers in oral squamous cell carcinoma [22]. IFNα has previously been reported to decrease the growth and metastasis of tumours [22]. It can activate the proliferation of immune cells and enhance the survival of T helper cells [22]. Furthermore, IFXα increases the number of tumour antigens expressed by tumour cells, allowing better detection by the immune system [22]. Meanwhile, CSCs were identified as a significant problem in regard to aiding cancer metastasis and chemoresistance [22]. They can initiate and enhance tumour survival by undergoing differentiation and self-renewal [22]. Although many therapies have been developed to target CSCs, the cells can avoid destruction by lying dormant [22]. Therefore, it is essential to investigate whether IFNα can be used to activate dormant CSCs and allow chemotherapeutic drugs to kill them [22]. 

**Figure 1:**
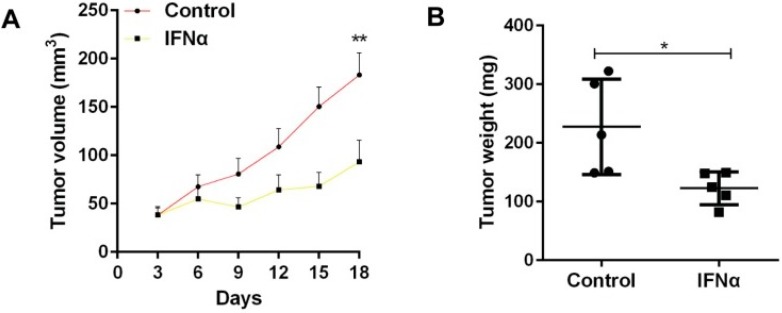
(**A**) The tumour volume was measured every three days. The administration of interferon-α (IFNα) decreased the rate of the tumour volume growth in comparison with the control group. ** represents the statistical significance (*p* < 0.01) of the difference in tumour volume between the two groups. (**B**) The tumour weight of each group was measured. The administration of IFNα significantly reduced the tumour weight in contrast with the control group (227.5 ± 36.4 vs 122.9 ± 12.5). * represents the statistical significance (*p* = 0.02) of the difference in tumour weight between the two groups [22].

Mice with tumours were divided into two groups: one that received IFNα and one that did not [22]. As shown in Figure 1, the administration of IFNα caused the resulting tumours to have significantly less weight and volume than that of the control [22]. The study also demonstrated how IFNα increased the expression of CSC markers, which allowed better identification of CSCs [22]. Overall, it was concluded that IFNα should be administered before using chemotherapeutic drugs to kill more cancer stem cells [22]. Like the previous experiment involving tumour necrosis factor-α, interleukin-1 and interleukin-6, the study showed that signalling molecules have the profound effect of inhibiting the growth of tumours [22]. This makes them valuable adjuvants that can enhance the strength of the immune system [22].

## 3. Inhibitors of Cancer Immune Evasion

Although there are many ways in which the immune system responds to cancer, there are also a plethora of methods that allow cancer cells to evade these defences. For example, cancer cells can vary the expression of their cell surface antigens, making it difficult for immune cells to recognise and destroy them [23]. Since the adaptive immune system targets specific antigens, it needs to be able to keep up with these changes and initiate responses against the new antigens if it is to succeed in combating cancer [24]. 

The host’s molecules can also aid the progression of cancer. Immune checkpoint proteins, which are naturally produced by the host in the maintenance of homeostasis, can also contribute to tumour formation [25]. These molecules aid in controlling immune responses and reducing the chance of inflammation and autoimmune diseases, thereby being crucial for self-tolerance [26]. However, tumours can utilise immune checkpoints to dampen immune responses. Therefore, researchers are discovering ways of inhibiting tumours’ ligand–receptor interactions via monoclonal antibodies that can act as immune checkpoint inhibitors [27]. Cytotoxic T-lymphocyte-associated antigen 4 (CTLA4) antibodies were the first to receive US Food and Drug Administration (FDA) approval [28]. Furthermore, anti-PD-1 targets programmed cell death protein 1 (PD1) [28]. CTLA4 and PD1 are immune checkpoints that can be present on the surface of T cells and interact with substances produced by tumour cells [28]. CTLA-4 controls T-cell division earlier on during immune responses, whereas PD-1 reduces this proliferation later [28]. Monoclonal antibodies can bind to these immune checkpoints to inhibit their functions and increase the activity of the immune system [28].

These inhibitors can be utilised in conjunction with radiation therapy [29]. Radiation therapy damages cancer cells with high doses of radiation targeted specifically at the area containing the tumour to prevent excessive damage to cells in other parts of the body [29]. This causes the spread of cancer cell antigens that can be detected by immune cells to stimulate a stronger response [30]. With the addition of checkpoint inhibitors, the immune system can attack tumours unrestrained by checkpoint molecules [31].

**Figure 2:**
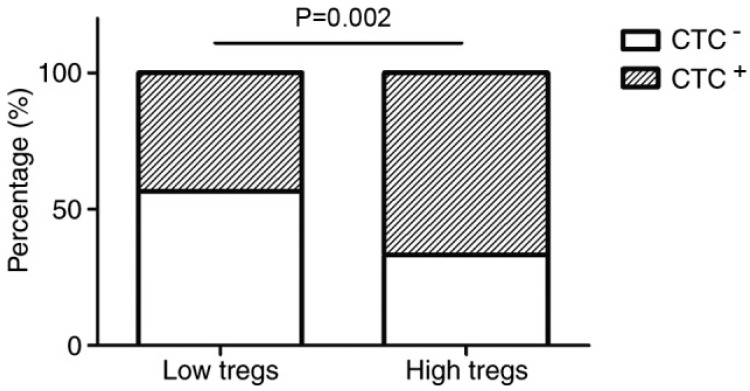
The relationship between regulatory T cells (Tregs) and circulating tumour cells (CTCs) in patients with breast cancer. The shaded region of the bars represents the percentage of individuals in the group that had CTCs. Individuals with a high number of Tregs were more likely to be CTC-positive than those that had a low number, and this result was significant (67% in contrast to 43%, *p* = 0.002) [34].

Additionally, therapies that target regulatory T cells (Tregs), which are part of the adaptive immune system, also need to be considered [32]. Tregs usually inhibit the functions of other T cells at the end of immune responses and prevent chronic inflammation from an overactive immune system [32]. Although they reduce the chance of inflammation-associated cancer development, evidence suggests that they may contribute to tumour survival [33]. A previous experiment was conducted on the relationship between Tregs and the dissemination of circulating tumour cells (CTCs) [34]. CTCs were involved in tumour metastasis and were present in 55% of breast cancer patients [34]. This is a significant issue, as metastasis allows the formation of secondary tumours in multiple areas and heightens the severity of cancer [34]. The results revealed a positive association between the number of Tregs, primary tumour size and the prevalence of CTCs [34]. Figure 2 shows that when there are a high number of Tregs, the percentage of individuals with CTCs is dramatically higher than that of the low Tregs group [34]. This affirms the belief that tumours can exploit Tregs, which aids their growth and spread [34]. Moreover, this emphasises that even in the presence of adjuvants, the actions of Tregs still need to be inhibited for effective cancer treatment [34]. 

Furthermore, cancer cells can produce galectins, which are proteins that induce the apoptosis of immune cells and contribute to tumour development [35]. Galectins contain carbohydrate recognition domains that enable them to bind to membrane-anchored cell surface receptors and trigger transmembrane signalling events, which are required for apoptosis to occur [36]. This is demonstrated in the ability of galectin-1 to target and destroy T cells, thus weakening cell-mediated immunity [37]. An experiment was performed to test this, whereby mice with lung cancer were exposed to either 10 mg/kg of saline or of thiodigalactoside every two days [38]. The number of T cells in the saline mice was significantly lower than that in the thiodigalactoside mice [38]. This is because the presence of thiodigalactoside resulted in a 50% reduction in galectin-1 levels, allowing more T cells to survive [38]. This reaffirms that decreasing levels of galectin-1 reduce the apoptosis of T cells and that the inhibition of galectin is a significant factor that needs to be considered in the treatment of cancer [38].

Galectin inhibitors can be combined with immune checkpoint inhibitors to produce more significant effects [39]. A previous experiment demonstrated how monoclonal antibodies could be combined with galectin inhibitors such as GR-MD-02, which inhibits extracellular galectin-3, to reduce the size of cancers [39]. This underscores the importance of synergism between various substances that target different aspects of cancer survival [39].

Nevertheless, major limitations exist in the production of galectin inhibitors [37]. The main problem is their inability to target intracellular galectins, causing progress to be slow [37]. Many galectins can traverse the plasma membrane of target cells and bind to intracellular compounds, evading current galectin inhibitors [37]. Thus, more evidence needs to be gathered regarding the actions and functions of intracellular galectins to find points along the reaction pathways that can be inhibited by new galectin inhibitors [37]. This will enable future treatments to target a wider variety of galectins and further reduce the apoptosis of immune cells to ensure stronger immunity [37].

## 4. The Advantages and limitations of Cancer Vaccines 

Generally, vaccines act by exposing the immune cells to antigens, whether they are free-floating or present on the surface of whole cells, and triggering an immune response [40]. This is an example of artificial active immunity and involves the differentiation and proliferation of immune cells [41]. Memory cells are also formed, providing immunological memory [42]. When exposed to the same antigen for the second time, the body can initiate a faster and stronger immune response due to the increased levels of memory cells and antibodies [43]. Both pathogens and cancer cells can be destroyed more efficiently [43]. However, vaccines used in isolation are not as effective as those combined with adjuvants [44]. Adjuvants are substances that modify the effects of vaccines and bolster immune responses to the antigens present [44]. This produces longer-lasting immunity and a stronger secondary response [44]. Adjuvants include cytokines, galectin inhibitors and immune checkpoint inhibitors [44].

Most vaccines aim to strengthen natural immune responses by exposing immune cells to antigens and increasing their proliferation and differentiation [45]. Autologous vaccines involve isolating cells from the body and then altering them before injecting them back into the patient’s body [45]. By using the individual’s cells, the chance of rejection by the immune system, which can occur when foreign antigens are detected, is decreased [45]. In the case of tumour cell vaccines, tumour cells processed in vitro are combined with an adjuvant to make them more easily detected and destroyed by the immune system [46]. 

DC vaccines expose DCs to cancer cells or antigens before they are reintroduced into the patient’s body, where they can more effectively attack cancer cells possessing the same antigens [47]. The actions of antigen-presenting cells are crucial in the initiation of specific immune responses against cancer [47]. An example of a DC vaccine is Provenge (Sipuleucel-T), which is used to treat prostate cancer [48]. Although it allows patients to survive longer with the disease, it is unable to cure it completely, and this is a major limitation that researchers are seeking to overcome [48]. 

DC vaccination was previously shown to be effective for CRC [49]. Patients that developed immunity after administration of the vaccine had better survival rates following surgery [49]. This increased survival was also positively associated with the T cell infiltration of CRC tumours [50,51]. A previous experiment aimed to investigate the relationship between vaccine responses and the densities of T cells in and near the tumour to recreate the results produced in the past by other researchers [49]. Immunohistochemistry allowed the number of T cells in various subpopulations to be determined [49]. No correlation was observed, which was different from the findings of previous research [49].

It was suggested that the effectiveness of the vaccine might have been altered in some way by somatic mutations in pathways involving tumour surface proteins and the PI3K/AKT/mTOR pathway [49]. The PI3K/AKT/mTOR intracellular signalling pathway is crucial in cell cycle regulation and plays a role in the longevity of cancer cells [52]. It is a convergence point for various upstream signalling molecules, such as apoptosis regulators and integrins [49]. Tumours would be able to maintain their size if they possessed reduced sensitivity to the compounds causing apoptosis [49]. In addition, integrins aid the metastasis and invasion of tumours [49]. Integrins are located on the cell surface and can interact with endothelial cells [49]. Metastatic CRC almost doubles the expression of integrin αVβ3 compared to nonmetastatic primary tumours, indicating the importance of integrins during this process [49]. Furthermore, cancer cells can also cause monocytes to release integrin αMβ2, allowing cell fusion and vascular migration [49]. The reduced concentration of integrin αMβ2 in monocytes can also hinder their development, resulting in less T cell priming following antigen presentation [49]. This can cause vaccine resistance and the reduced infiltration of T cells [49]. Therefore, PI3K inhibitors and integrin inhibitors are especially effective in targeting metastatic cancer, as demonstrated in several clinical trials [49]. 

Nevertheless, the study only included 22 participants with CRC and liver metastases [49]. The small sample size made it more difficult to achieve reliable results, thus proving to be a significant limitation [49]. The previous studies utilised large sample sizes to assess the relationship between survival rates and T cell infiltration [50,51]. This may also contribute to Qian et al. being unable to replicate the results of their predecessors. 

Furthermore, another DC vaccine for CRC exists, which includes tumour lysates [53]. CD14+ monocytes are differentiated into DCs via exposure to GM-CSF and IL-4 [53]. This is followed by incubation with autologous tumour lysate and activation of the DCs by a variety of compounds, including interferons and tumour necrosis factor-alpha [53].

Cancer vaccines have been used to prevent the relapse of patients suffering from CRCs. These include combining autologous tumour cells with bacillus Calmette Guerin (BCG) [53]. However, while this worked for stage II CRCs, it did not work for stage III or IV CRCs [53]. Nevertheless, the new dendritic vaccine improved disease-free survival (DFS), the length of time after treatment that a patient lives without cancer [53]. A suggestion to further improve outcomes is the addition of checkpoint inhibitors [53]. Anti PD-1 or PD-L1 can be administered as adjuvants before surgery, which would increase the strength of immune responses once DC vaccinations occur after surgery [53]. Equally, many previous studies recommend the use of adjuvant chemotherapy for the reduction of relapse risk [54]. 

A limitation of this study is that it only contained 15 randomised patients due to logistical and financial problems [53]. Despite this, the patients were still followed up as initially planned to evaluate their relapses [53]. These individuals received adjuvant chemotherapy before randomisation occurred [53]. Similarly to the study by Qian et al., the small sample size may have adversely affected the results [53]. However, the researchers are optimistic about the potential benefits of DC vaccines that have been combined with autologous tumour lysates [53].

In contrast to whole cell vaccines, antigen vaccines do not utilise the entire tumour cell [55]. They only contain the antigens on the cell that are most effective in eliciting an immune response [55].

Allogenic whole cell vaccines can be used for various individuals as they are not specific for each patient, unlike autologous vaccines [56]. This allows them to be easier to manufacture, thus saving both time and money [56]. An example of an allogeneic whole cell vaccine is Canvaxin, which is made up of melanoma antigens from different cell lines and patients [57]. In the experiment, the cell lines were irradiated to prevent cell division, before being kept at low temperatures to ensure their preservation [57]. From there, the cells can be thawed and injected into patients when required [57]. It was demonstrated that vaccinated individuals had a median survival period of greater than 53 months, whereas those unvaccinated had around 42 months [57]. At the end of the five years, 50% of the vaccinated group were alive, compared to only 25% of the unvaccinated group [57]. This elucidates the effectiveness of this vaccine in reducing the impacts of cancer [57]. Importantly, the method employed in the production of Canvaxin was essential to furthering the development of more cancer vaccines [57].

One of the significant limitations concerning cancer vaccines is the low approval rate [58]. Provenge is the only one that has been approved by the FDA [58]. This has resulted in a substantial number of trials remaining in the phase 2 stage and unable to advance to phase 3 [58]. The main reason why there is such a low approval rate is that the system used to assess cancer vaccines is outdated and more suited to testing conventional chemotherapeutic agents [58]. Cancer vaccines can cause an increase in tumour burden since more cancer cells are initially introduced into the body, but this only lasts for a short period of time before the immune system is strengthened and can fight back [58].

Additionally, the effects of these vaccines are not as immediate as traditional treatment methods such as radiation therapy, due to the time required for the immune system to detect antigens and initiate a response [58]. Numerous processes are involved, including phagocytosis, antigen presentation and lymphocyte differentiation and proliferation [58]. Additionally, many cancer vaccines are made with tumour antigens that are too similar to those on healthy cells [58]. To prevent the destruction of healthy cells, the body weakens the immune response against cancer [58]. However, this enables abnormal cells to grow more rapidly [58].

These factors can all contribute to the imbalance between the perceived view of cancer vaccines and their actual effectiveness, which hinders their acceptance by both the FDA and the wider community [58]. Furthermore, as mentioned in Topic 2, the array of methods by which tumours can evade and suppress the immune system create difficulties in their treatment, even with the aid of immunotherapy [23]. However, while the number of trials involving only cancer vaccines has remained constant over the past two decades, trials experimenting on adjuvants in combination with traditional vaccines have increased dramatically due to researchers gaining more knowledge on their importance in cancer treatment [58]. 

It is crucial to develop more cancer vaccines since they have low toxicity and do not possess as many side effects as other treatment options, such as radiation therapy and chemotherapy [43]. Although treatment with checkpoint inhibitors and radiation therapy can be highly effective, there are numerous side effects associated with radiation therapy [59]. These include a dry and sore mouth, nausea, tooth decay, as well as lymphedema, which is a form of swelling that occurs in arms and legs due to damaged lymph nodes [59]. Equally, while chemotherapy can target cancer cells throughout the body through its use of circulating anti-cancer drugs, it can cause the death of healthy rapidly dividing cells, such as hair and skin cells, and result in hair loss and easy bruising [60]. These side effects highlight the importance of continuing cancer vaccine research.

## 5. Adjuvants and Cancer Vaccines

By understanding the principles underlying the previous topics of discussion, researchers can utilise this knowledge to strengthen cancer vaccines. These methods show great promise for the next generation of cancer treatment due to their minimal side effects in comparison to chemotherapy and ionising radiation [60]. 

Cytokines, such as the granulocyte-macrophage colony-stimulating factor (GM-CSF), are major adjuvants that have been commercialised [61]. GM-CSF is a white blood cell growth factor and is released by a variety of immune cells such as macrophages and T cells [61]. GVAX, produced by the company Cell Genesys, is an allogenic cancer vaccine that contains GM-CSF [61]. It is created by exposing a mixture of interferons to tumour cells taken from patients, which enhances the expression of B7-1 proteins on their cell surface and strengthens the vaccine [61]. B7-1 proteins are usually found on the surface of antigen-presenting cells and bind to proteins on T cells to produce signals that can stimulate them [61]. Moreover, the tumour cells are genetically modified to release GM-CSF, which can increase the activity of the immune system [61]. They are then irradiated to prevent cell division, before being reinjected into the patient [61]. Previous experiments were performed on mice to test the effectiveness of this vaccine [61]. When the vaccine was reinjected into mice, there was a significant increase in the number of cytotoxic T cells, which enabled the destruction of tumours [61].

Equally, another experiment attempted to measure the effects of increased B7-1 expression in allogenic vaccines but took it a step further by also incorporating 4-1BBL ligands [62]. Through exposure to various interferons, four groups of cancer cells were produced: a control group, those that overexpressed B7-1, those that overexpressed 4-1BBL, and those that overexpressed both ligands [62]. These cells were used to vaccinate the mice, and the data of the memory T cell populations were collected from the different areas of the circulatory and lymphatic systems via flow cytometry [62]. The cancer cells were able to mimic the action of antigen-presenting cells and stimulate cytotoxic T cells, and the presence of both ligands aided the recognition process, thus producing a stronger response than that of cancer cells with only one or no ligands [62]. The presence of DCs was not necessary since mice with low concentrations of these cells were still able to be affected by the vaccine [62]. Nevertheless, it was noted that increasing the number of these cells still aided in bolstering the action of the vaccine due to the role of DCs as antigen-presenting cells necessary for the initiation of cell-mediated immunity [62]. Ultimately, this research demonstrates the importance of interferons as adjuvants in increasing the detectability of cancer, similarly to the previous experiment involving GM-CSF [62]. It also reiterates that allogeneic whole cell vaccines can be an effective treatment that is faster, cheaper and can be administered to a broader range of patients than the commonly used DC vaccines [62].

Furthermore, monoclonal antibodies and galectin inhibitors can also be utilised as adjuvants, and evidence suggests that their combined use with vaccines produces a synergistic effect [39]. Two phase 1 clinical trials were conducted on patients with varying types of cancer, including head and neck squamous-cell carcinoma and metastatic melanoma [39]. Ipilimumab (anti-CTLA-4) or pembrolizumab (anti-PD-1) were combined with galectin inhibitor GR-MD-02 and administered to patients [39]. The combination of immune checkpoint inhibitors and galectin inhibitors allowed more effective treatment of patients compared to using the substances in isolation [39]. These results reflect murine tumour models, where it was found that mice exposed to the broadest range of antitumor compounds experienced the most considerable reduction in tumour growth [39].

The various experiments presented in this review demonstrate how synergism can be utilised to achieve higher outcomes. However, none accomplished as much success as a newly developed cancer vaccine that resulted in a 100% survival rate in mice [63]. An experiment sought to investigate the effectiveness of Diprovocim as an adjuvant by comparing it to anti-PD-L1 and alum, an aluminium salt which is one of the most widely used adjuvants [63]. The cancer vaccine tested was one that immunised mice against ovalbumin, a tumour-specific antigen [63]. There were three groups of mice being experimented on: one that was exposed to the cancer vaccine and anti-PD-L1, one that was also presented to alum, and one that was exposed to Diprovocim instead of alum [63]. 

**Figure 3:**
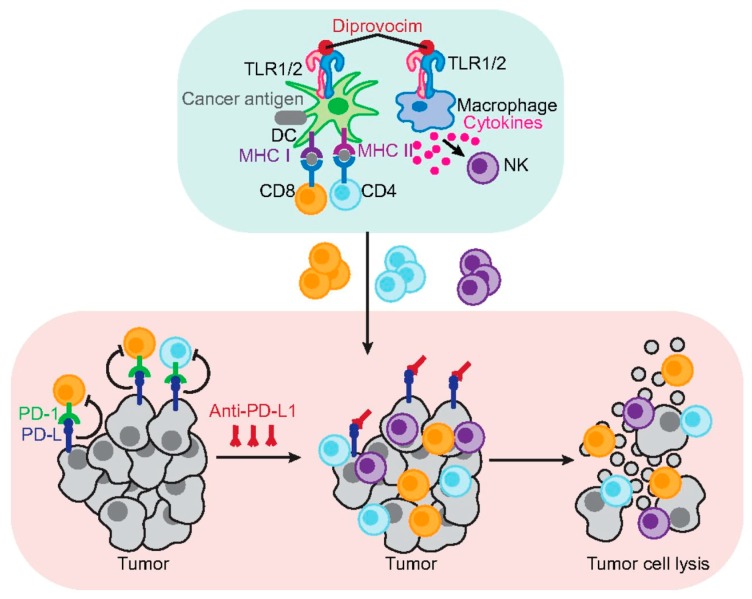
Diprovocim (TLR1/TLR2 agonist) acts in combination with anti-PD-L1 to cause tumour cell lysis, leading to a 100% survival rate in mice. Anti-PD-L1 decreases the inhibition of immune cells to allow an unrestrained response against tumours. Diprovocim increases antigen presentation and cytokine release, increasing immune cell proliferation [63].

The results showcased that the first group had no survivors, while one-quarter of the mice in the second group survived, demonstrating the effectiveness of alum in boosting survival rates [63]. However, this paled in comparison to the action of Diprovocim, which produced a curative response in the treatment of melanoma [63]. As Figure 3 shows, Diprovocim can activate antigen-presenting cells by binding to their surface receptors and increasing antigen presentation and cytokine release [63]. This increases the number of tumour-specific cytotoxic T cells and helper T cells which, in conjunction with immune checkpoint inhibitors, can have an unrestrained response against cancer cells to cause tumour cell lysis [63]. These findings not only explicate the effectiveness of Diprovocim, but also demonstrate how crucial synergism is to the success of cancer vaccines [63].

Furthermore, the co-encapsulation of phosphorylated adjuvants and cancer antigens was proposed as a potential method to treat CRCs that had spread to the liver [12]. The encapsulation was performed by the lipid calcium phosphate nanoparticle, allowing the production of a vaccine carrier [12]. The phosphorylated adjuvants included were 2′3′cGAMP, CpG, and 5′pppdsRNA [12]. The cancer antigen was a peptide called p-AH1-A5 [12]. Compared to other adjuvants and unvaccinated controls, this specific combination of adjuvants and antigens was more effective in reducing the growth and metastasis of tumours [12]. In particular, 5′pppdsRNA was able to increase the number of CD8+ T cells without increasing regulatory T cells [12]. This allowed a stronger immune response without extra hindrance from immune suppressors [12]. 

Equally, the adjuvant CpG was utilised in conjunction with interleukin−2 (IL−2) in a DNA vaccine in order to study immune responses against CRCs in mice [64]. CpG sites are regions in the DNA where cytosine nucleotides are followed by guanine nucleotides [64]. They are often found in CpG islands, where methylation can occur to decrease the transcription of genes and thus reduce protein production [64]. A bicistronic plasmid containing a CpG motif was utilised to carry the genes of IL-2 and survivin/MUC1 (MS) [64]. Bicistronic refers to the presence of two cistrons, which allows bicistronic transcription [64]. CpG increased the cytotoxicity of the vaccine by a factor of 5, and the metastatic tumour foci were suppressed by 69.1% [64]. Survivin and MUC1 generated immune responses in splenocytes [64]. This increased the expression of compounds such as GM-CSF and CCL-19, while decreasing the production of immune-suppressing factors, including PD-L1 [64]. Similarly to the experiment by Goodwin and Huang, the vaccine increased the strength of the immune response to cancer and decreased immune suppression [12]. Moreover, the addition of the chemotherapy drug oxaliplatin lengthened the lives of the mice by 2.5-fold, suggesting that the combination of chemotherapy and cancer vaccines can allow better defence against CRCs [64].

Overall, this literature review is essential to the theoretical framework since it demonstrates how combinations of different adjuvants can be utilised in a variety of cases to enhance the effects of cancer vaccines. This review evaluated the effectiveness of cytokines, galectin inhibitors, phosphorylated adjuvants and monoclonal antibodies, both in isolation and in conjunction with other substances. Several of these adjuvants have been utilised in the treatment of CRC. Earlier research was presented, such as the experiment conducted on the effectiveness of the cytokine tumour necrosis factor-α, interleukin-1 and interleukin-6. This showcased previous studies and knowledge regarding cancer treatment. In addition, newer experiments, including some involving Diprovocim and IFNα, were also discussed. This not only allows comparisons to be made with older publications but also illustrates recent advances in this field. It offers hope to patients that new successful vaccines may be able to be approved by the FDA and potentially be used by millions of people to treat CRC.

## 6. Conclusions

Ultimately, the concept that cancer is not a single disease but rather a group of diseases needs to be acknowledged before any significant progress can be made. A magic bullet does not exist, as one treatment does not work on all types of different cancers. Thus, this literature review emphasises the importance of personalised immunotherapy, which can produce a targeted response against CRCs. This study has achieved the aim of showcasing the current status of cancer vaccines and the further improvements that can be made through the utilisation of adjuvants. 

Regarding future prospects, ongoing research is likely to be conducted on vaccines containing antigenic compounds that specifically correspond with those on the cancer cells of patients. Furthermore, methods to counteract their major limitations should be investigated. As mentioned previously, cancer vaccines have low approval rates due to the long time it takes for them to exert their effects in the body thoroughly. By developing combination therapies, adjuvants can aid in controlling the growth of tumours and provide the necessary time for antigen presentation and white blood cell proliferation to occur. As discussed in numerous experiments in this literature review, synergism is crucial. Suitable adjuvants, including cytokines, galectin inhibitors, phosphorylated adjuvants, monoclonal antibodies and Diprovocim, should be combined to produce greater effects than if they were administered in isolation. This supports the hypothesis that adjuvants, when combined with cancer vaccines, significantly enhance the immune system’s ability to fight cancer synergistically. 

As mentioned previously, vaccines containing tumour antigens too similar to those on healthy cells could cause the weakening of immune responses to avoid destroying healthy cells. Thus, it would be beneficial to utilise tumour antigens that can be easily differentiated from those on normal cells. The methods detailed above serve to reduce the limitations of cancer vaccines and offer an ideal view of the future. Overall, with rapid advancements in technology and knowledge, the prospect of one day developing personalised CRC therapies is more likely than ever before. 

## Abbreviations

CRC	Colorectal carcinoma
MHC	Major histocompatibility complex
DC	Dendritic cell
IFN	Interferon
CSC	Cancer stem cell
Treg	Regulatory T cell
FDA	US Food and Drug Administration
CTLA4	Cytotoxic T-lymphocyte-associated antigen 4
PD1	Programmed cell death protein 1
CTC	Circulating tumour cell
BCG	Bacillus Calmette Guerin
DFS	Disease-free survival
GM-CSF	Granulocyte-macrophage colony-stimulating factor
IL	Interleukin
MS	Survivin/MUC1
